# Safety and Immunogenicity of *Morbillivirus canis* Vaccines for Domestic and Wild Animals: A Scoping Review

**DOI:** 10.3390/v16071078

**Published:** 2024-07-04

**Authors:** Santiago Rendon-Marin, Luis Felipe Higuita-Gutiérrez, Julian Ruiz-Saenz

**Affiliations:** 1 Grupo de Investigación en Ciencias Animales—GRICA, Facultad de Medicina Veterinaria y Zootecnia, Universidad Cooperativa de Colombia, Bucaramanga 680001, Colombia; santiago.rendon@campusucc.edu.co; 2 Grupo de Investigación Infettare, Facultad de Medicina, Universidad Cooperativa de Colombia, Medellín 050001, Colombia; 3 Facultad de Medicina, Universidad Cooperativa de Colombia, Medellín 050001, Colombia; luis.higuita@campusucc.edu.co; 4 Escuela de Microbiología, Universidad de Antioquia, Medellín 050001, Colombia

**Keywords:** *Morbillivirus canis*, vaccination, domestic animals, wildlife, scoping review

## Abstract

*Morbillivirus canis* (canine distemper virus (CDV)) is recognized as a multihost pathogen responsible for a transmissible disease affecting both domestic and wild animals. A considerable portion of wildlife populations remain unvaccinated due to a lack of safety and immunogenicity data on existing vaccines for the prevention of CDV infection in these species. This review aimed to assess the current state of CDV vaccination research for both domestic and wild animals and to explore novel vaccine candidates through *in vivo* studies. It also sought to synthesize the scattered information from the extensive scientific literature on CDV vaccine research, identify key researchers in the field, and highlight areas where research on CDV vaccination is lacking. A scoping review was conducted across four databases following the PRISMA-ScR protocol, with information analyzed using absolute and relative frequencies and 95% confidence intervals (CIs) for study number proportions. Among the 2321 articles retrieved, 68 met the inclusion criteria and focused on CDV vaccines in various animal species, such as dogs, ferrets, minks, and mice. Most of the scientific community involved in this research was in the USA, Canada, France, and Denmark. Various vaccine types, including MLV CDV, recombinant virus, DNA plasmids, inactivated CDV, and MLV measles virus (MeV), were identified, along with diverse immunization routes and schedules employed in experimental and commercial vaccines. Safety and efficacy data were summarized. Notably, 37 studies reported postimmunization CDV challenge, primarily in dogs, revealing the survival rates of vaccinated animals. In summary, CDV vaccines generally demonstrate an acceptable safety profile in dogs and show promise as a means of controlling CDV. However, significant gaps in vaccine research persist, particularly concerning wildlife reservoirs, indicating the need for further investigation.

## 1. Introduction

*Morbillivirus canis*, commonly known as canine distemper virus (CDV), causes a highly contagious disease in domestic and wildlife animals, and CDV infection has been reported in more than 8 orders and 20 families worldwide [1]. The host range of CDV predominantly includes species within the order Carnivora, spanning diverse families such as *Canidae Procyonidae*, *Mustelidae*, *Ursidae*, *Ailuridae*, and *Felidae*, and, to a lesser extent, other notable families from different orders, namely *Artiodactyla*, *Primates*, *Rodentia*, and *Proboscidea* [2,3,4]. Given the extensive spectrum of species affected by CDV, different studies have explored its cross-species transmission among both wild and domesticated animals, investigating not only their interactions to elucidate the molecular diversity but also strategies such as vaccination to control CDV’s spread in domestic and wild animals [1,5].

Canine distemper disease (CDD) manifests with a range of clinical symptoms, such as fever, as well as respiratory, gastrointestinal, and neurological issues, mainly in domestic dogs [6]. The clinical signs in wildlife are mainly associated with neurological disorders [7]. CDV is included in the *Paramyxoviridae* family, genus *Morbillivirus*, and has a single-stranded negative-sense RNA genome with six transcription units that encode for eight proteins [1]. Among these proteins, the H and F proteins are essential, because of their function in diverse essential viral processes [8]. Both proteins are involved as the main antigenic determinants of CDV due to their viral particle localization [9]. Moreover, they have been employed in CDV vaccine alternatives [10], helper T-cell epitopes from the F protein have been identified [11], and, finally, H and F peptides have been recovered in higher proportions than other viral proteins from dog major histocompatibility complex (MHC) molecules [12].

The H protein is considered the most variable protein among all lineages [13], and, to date, based on its entire sequence, diverse genotypes have been described, such as America-1, America-2 to -5, Arctic-like, Rockborn-like, Asia-1 to -4, Africa-1 to -2, European Wildlife, Europe/South America-1, South America-2 and -3, and South/North America-4 [14,15]. The re-emergence of different CDV infections in domestic and wildlife animals has demonstrated the importance of updating the vaccine strains and examining the status of CDV vaccination, since commercial vaccines are not fully employed in wildlife animals due to the lack of relevant evidence of their safety and efficacy [16]. Diverse circulating lineages, geographically distributed with amino acid changes in viral proteins involved in the host immune response and viral neutralization, could explain the vaccine failure in response to wild-type emerging strains, cross-species transmission, and the increased virulence of emerging CDV strains in domestic dogs and wildlife animals [17]. Additionally, case reports associated with CDV infection in wild animals have recently been published [3], with CDV being a threat to a wide range of in-danger species [16,18].

The transmission dynamics of CDV between domestic and wild animals are not fully understood. Therefore, examining these transmission dynamics and identifying CDV reservoirs holds significant promise for the formulation of effective strategies to safeguard endangered species [17]. Some reports suggest that vaccinating wildlife may play a pivotal role in preventing the extinction of at-risk populations due to CDV [5,18]. Thus, immunizing animals and ensuring safety in both domestic and wildlife populations represent a field of study that warrants extensive efforts to ensure the health of companion animals and those endangered by CDV infection.

Regarding the current state of vaccination, MLV vaccines based on traditional America-1 strains (i.e., the Onderstepoort strain) and Snyder Hill, Convac, Rockborn, or CDV3 strains [19] are commercially available. However, these strains no longer circulate around the world [20]. Recombinant CDV vaccines have been developed by integrating the CDV F and H proteins into a canarypox virus vector, and these vaccines have demonstrated a safe profile across various susceptible species, including dogs, European ferrets (*Mustela putorius furo*), giant pandas (*Ailuropoda melanoleuca*), fennec foxes, meerkats (*Suricata suricatta*), and Siberian polecats (*Mustela eversmanni*) [21,22,23,24]. Although recombinant canarypox vaccines exhibit safety and moderate efficacy across a spectrum of species, they induce a less robust immune response than MLV vaccines, primarily due to replicative limitations [25]. However, this characteristic underscores their suitability for the immunization of young dogs in the presence of maternal antibodies [9]. Nevertheless, it has become imperative not only to develop new vaccine strategies to control CDV infection but also to assess the efficacy and safety of the currently available vaccines, since there is limited experimental evidence regarding these vaccines in wildlife animals. Other alternative strategies include experimental assays, including a recombinant bivalent vaccine, which employs a rabies virus that expresses the H and F CDV proteins, evaluated in domestic dogs and ferrets [19].

Based on the limited spectrum of current vaccines, different researchers worldwide have investigated new vaccines against CDV through *in vivo* studies involving diverse animal species, such as domestic dogs, BALB/c mice, minks, and ferrets, including recombinant viruses [26,27,28,29], chimeric measles virus constructs expressing CDV proteins [30], DNA vaccines encoding the main antigenic determinants of CDV [31,32,33], the H and F CDV proteins isolated as antigens [34], recombinant mouse adenovirus 1 expressing CDV antigens [35], and a novel vaccine formulation based on bacterium-like particles presenting CDV antigens [19]. However, the state of CDV vaccination research, development, and efficacy evaluation have yet to be explored and summarized in order to establish different vaccination schemes to control CDV’s transmission, dissemination, and disease course.

Therefore, this scoping review aims to assess the current state of research and advancements in CDV vaccination for both domestic and wild animals. This review encompasses the evaluation of existing vaccines in various species and the investigation of novel vaccine candidates through *in vivo* studies. Given the proliferation of reports on new vaccination strategies for CDV, this inquiry is warranted to target both wildlife and domestic canine populations. Furthermore, this scoping review seeks to consolidate the fragmented information scattered throughout the extensive scientific literature regarding CDV vaccine research. Additionally, this study aims to identify key researchers in this field and pinpoint regions where research on CDV vaccination is lacking.

## 2. Materials and Methods

### 2.1. Type of Study

Following the recommendations of the PRISMA-ScR [36] and the Joanna Briggs Institute, a systematic scoping review on CDV vaccination and development for domestic and wildlife animals was conducted. The scoping review was registered at https://osf.io/n9sed (accessed on 22 May 2024) and the PRISMA-ScR checklist is available in Table S1.

### 2.2. Search and Study Selection

The data were searched in multidisciplinary repositories such as PubMed, SciELO, ScienceDirect, and Scopus. For term selection, we used the Descriptors in Health Sciences (DeCS), Medical Subject Heading (MeSH), and both known names for CDV, with truncated terms vacc* AND canine distemper virus and vacc* AND canine morbillivirus, in all databases. A total of 8 searches were performed as follows: SciELO: (ti:(canine distemper virus)) AND (ti:(vacc*)) AND (ab:(canine distemper virus)) AND (ab:(vacc*)) and (ti:(canine morbillivirus)) AND (ti:(vacc*)) AND (ab:(canine morbillivirus)) AND (ab:(vacc*)); Scopus: (TITLE-ABS-KEY (canine AND distemper AND virus) AND TITLE-ABS-KEY (vacc*)) and (TITLE-ABS-KEY (canine AND morbillivirus) AND TITLE-ABS-KEY (vacc*)); PubMed: (vacc*[Title/Abstract]) AND (canine distemper virus[Title/Abstract]) AND (vacc*[Title/Abstract]) AND (canine morbillivirus[Title/Abstract]); and, finally, ScienceDirect: canine distemper virus AND vacc? and canine morbillivirus AND vacc?. The articles retrieved from each search (restricted to title, abstract, and/or keywords) were saved in a common file in Zotero to eliminate duplicates.

### 2.3. Screening and Eligibility

We screened studies published historically until 2024 (the last update was performed on 9 January 2024), including original research (eliminating reviews, editorials, protocols, and book chapters) that was conducted on CDV vaccination and development for domestic and wildlife animals *in vivo*, and the language was also considered (including English, Spanish, and Portuguese). These criteria were applied by two researchers independently. During the eligibility phase, studies were excluded if they did not include safety information, such as weight changes, temperature, survival, the immunization route, or efficacy, measured as either specific or neutralizing CDV antibodies.

### 2.4. Data Extraction

The following variables were extracted from the selected studies: title; authors; year of publication; country of publication; sample size; species; immunogen type; immunization route; vaccine safety measured by temperature, weight, behavior, leukocyte count, and survival; and efficacy through specific antibody (Ab) production measured either with neutralization assays or with ELISA. In studies where viral challenge was reported, the inoculation route and challenge survival were also retrieved. All information regarding the included studies was synthesized in figures and tables.

### 2.5. Data Charting Process

The data charting process was independently developed by two researchers to ensure concordance across all phases of the search, study selection, and data extraction processes. A third investigator resolved any discrepancies through consensus and further examination.

### 2.6. Analysis of the Information

The description of different aspects of the included studies was performed with absolute and relative frequencies and 95% confidence intervals (CIs). A scientific collaboration network in this research field was created with Gephi. The difference in survival between vaccinated and nonvaccinated animals was calculated using the Z test or confidence interval for the difference in proportions. The frequency analyses and Z tests were performed with EPIDAT version 4.2.

## 3. Results

### 3.1. Study Selection and Prisma Flow Chart

**Figure 1:**
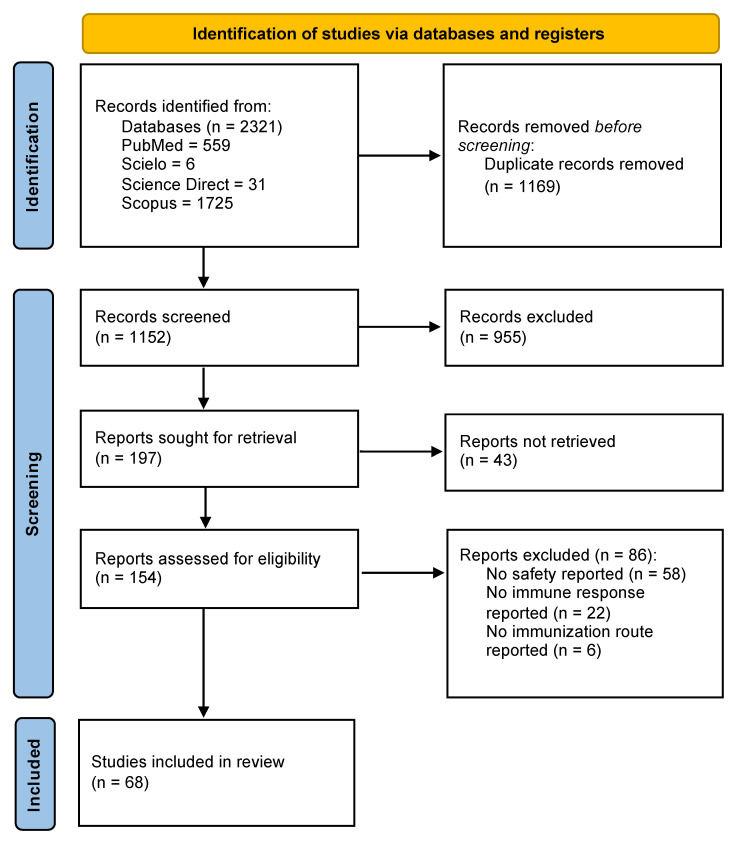
Study selection algorithm based on PRISMA guidelines.

Initially, 2321 studies were identified in PubMed, SciELO, Science Direct, and Scopus. After duplicates were removed, the number of articles for screening decreased to 1552. Then, the inclusion criteria were applied, and 154 studies were included. Finally, 68 studies were included in this scoping review after the application of the exclusion criteria (Figure 1).

### 3.2. International Cooperation and Researchers

**Figure 2:**
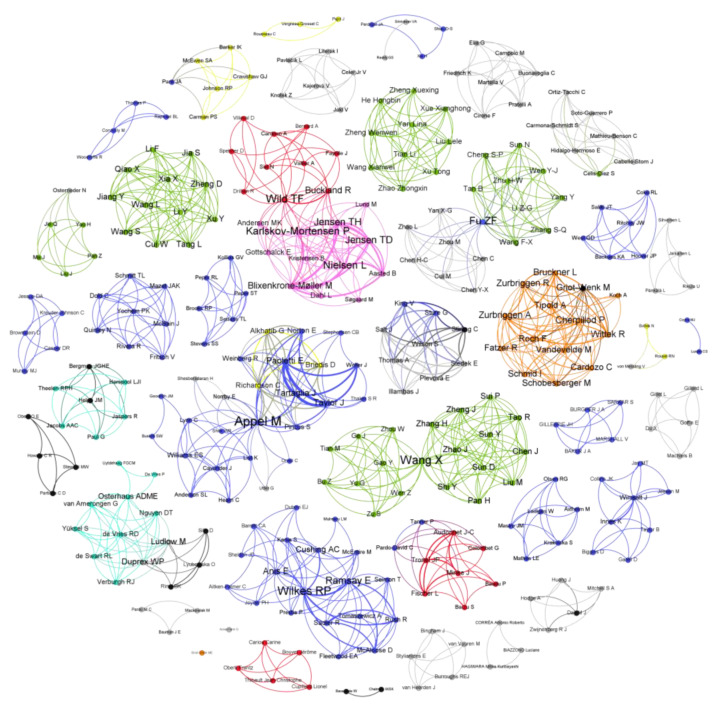
Scientific collaboration network related to research on CDV vaccination development. Relationships among researchers based on country of affiliation. The United States of America is marked in blue, China in green, France in red, the Netherlands in cyan, Switzerland in orange, Canada in yellow, the United Kingdom in black, and Denmark in pink. Low-frequency countries are marked in gray. The diameter of each node denotes the importance of this researcher in CDV vaccine development. The graph was created with Gephi.

To understand the level of international cooperation in the development of CDV vaccines, a scientific collaboration network analysis of researchers was carried out, considering their countries of affiliation. The scientific community related to CDV vaccination in domestic and wildlife animals comprised 308 researchers from diverse countries, including the United States of America, China, France, the Netherlands, Switzerland, Canada, the United Kingdom, and Denmark (Figure 2). Notably, there are few researchers from different countries who collaborate in CDV vaccine research, as evidenced by the presence of mostly individual nodes in the collaboration networks shown in Figure 2. Collaboration was evidenced only between researchers from the USA and Canada and between researchers from France and Denmark. In the figure, the size of the author’s name denotes the importance of CDV vaccine development since it represents the presence of these authors in different studies. Among the authors who have contributed greatly to CDV vaccination research, in the USA, Rebecca Wilkes has performed different studies in wild animals, and Max J.G. Appel contributed to initial MLV CDV vaccines in dogs, especially those based on the Onderstepoort strain. In France, T. Fabian Wild has also contributed considerably, including studies in mice and minks, employing recombinant and DNA vaccines (Table 1).

### 3.3. Included Studies and Countries Contributing to CDV Vaccine Development

A total of 68 studies were included in the scoping review. The main characteristics of the included articles are summarized in Table 1. The geographic distribution included studies from the United States of America (*n* = 31); China (*n* = 7); France (*n* = 5); Belgium, Canada, Denmark, the Netherlands, and the United Kingdom (*n* = 3 in each country); Switzerland (*n* = 2); and Australia, Brazil, the Czech Republic, Finland, Germany, India, Italy, and South Africa (*n* = 1 in each country). The United States of America had the greatest number of *in vivo* CDV vaccine studies, followed by China and France, which correlates with the authors’ contributions as reported in Figure 2.

### 3.4. Domestic and Wild Animals in CDV Vaccine Trials

As shown in Table 2, 37.3% of the studies were carried out in dogs, followed by ferrets, mice, minks, African wild dogs, tigers, and cats, with only one study on other species (Table 2), shown as a relative frequency (RF). A total of 2363 individuals were included in all studies on diverse animal species; however, considering the vast array of species affected by CDV, there is still a lack of *in vivo* CDV vaccine studies focused on the control of CDD and its dissemination among domestic and wildlife animals, due to the lower number of studies corresponding to either domestic or laboratory animals.

### 3.5. CDV Vaccine Characteristics in Included Stidies

The different immunogenic approaches employed in the included studies and their characteristics are summarized in Table 3. The most frequent vaccine types were MLV CDV (*n* = 25) and recombinant virus (*n* = 25), with the same number of studies, as well as DNA plasmids (*n* = 13), inactivated CDV (*n* = 6), MLV MeV (*n* = 3), and others (*n* = 4). On the other hand, different immunization schemes were employed with one (*n* = 25), two (*n* = 31), three (*n* = 16), and four (*n* = 5) doses (Table 3), where the two-dose scheme had the highest percentage, at 40.3% of the total number of studies. Moreover, regarding the inoculation route, most studies employed subcutaneous immunization (*n* = 36), followed by intramuscular (*n* = 30), oral (*n* = 6), and other methods (*n* = 11).

### 3.6. Safety and Efficacy of CDV Vaccines from Included Studies

The main characteristics of vaccines are safety and efficacy. Although there are diverse alternatives to evaluate safety, in the included studies, the clinical signs (*n* = 65), temperature (*n* = 26), weight loss (*n* = 21), survival (*n* = 14), animal behaviors (*n* = 6), and leucocyte counts (*n* = 5) were the primary indicators. In addition, neutralizing antibodies (*n* = 66) and specific antibodies such as IgG (*n* = 15), detected by ELISA, were reported as measures of the humoral immune response to immunization with CDV vaccines (Table 4). Overall, 54.4% of the included studies involved infectious virus challenge (*n* = 37) with different CDV lineages, and intranasal challenge was the most common route (*n* = 22), followed by intravenous challenge (*n* = 2) and other methods (*n* = 17), as shown in Table 4.

As mentioned before, a total of thirty-seven studies reported postimmunization virus challenge (Table 4) on different species. Seventeen studies included dogs, eight included ferrets, five included minks, four included mice, one study included foxes, one included raccoons, one included Siberian polecats, and one included hybrid ferret WITH Siberian polecats. One study included two animal populations, mice and foxes, totaling thirty-eight animal populations.

### 3.7. Postimmunization Challenge and Efficacy for CDV Vaccine Trials

**Figure 3:**
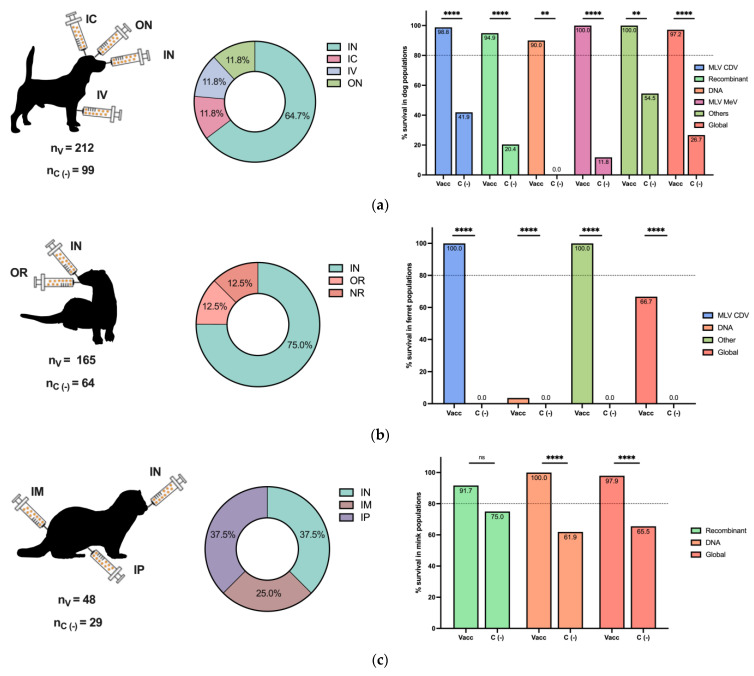

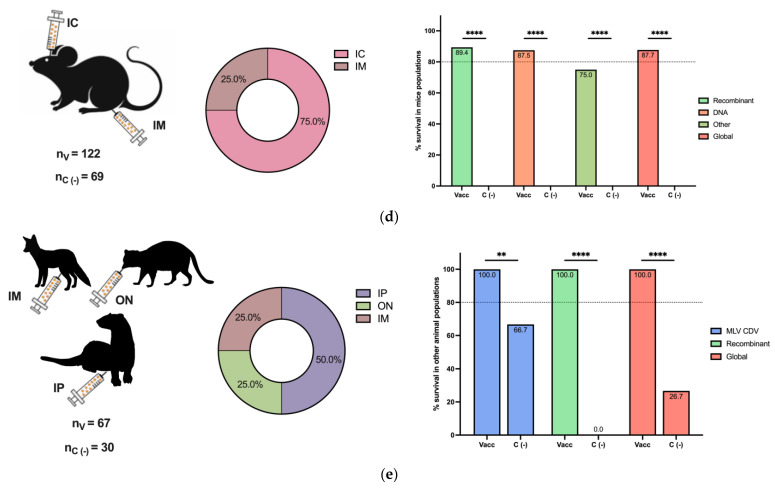
Postimmunization CDV challenge in different animal populations. The numbers of included vaccinated (n_V_) and negative vaccination control (n_c (−)_) animals of all challenged species are reported. The inoculation routes are shown, as well as the percentage of survival based on the vaccine type for (**a**) dog populations, (**b**) ferret populations, (**c**) mink populations, and (**d**) mouse populations. (**e**) Other populations, such as foxes, raccoons, Siberian polecats, and hybrid ferrets WITH Siberian polecats, are described. Other vaccines included MLV MeV, inactivated CDV, ISCOMs, and purified H and F CDV proteins in dogs; chimeric MeV-expressing CDV antigens in ferrets; and peptide-based vaccines in mice. IN: intranasal, IC: intracranial, SC: subcutaneous, IP: intraperitoneal, IM: intramuscular, ON: oculonasal, IV: intravenous, OR: oral, ID: intradermal, IDU: intraduodenal, NR: not reported. Z test for the difference in proportions with ** *p* < 0.01, **** *p* < 0.0001 and ns: not significance.

The efficacy of the CDV challenge is reported in Figure 3 for different animal populations. As expected, the dog was the most common animal included in vaccine and challenge studies, followed by ferrets and mice (Figure 3a–e, left panels). Intranasal, oral, and oronasal CDV inoculation was broadly employed in dogs, ferrets, and minks (Figure 3a–c, middle panels), since this route could simulate a natural CDV infection, in contrast to other routes, such as IM, IC, and IP, among others, used for mice and other animals (Figure 3d,e, middle panels).

For each animal species, the overall survival percentage was evaluated as the cumulative survival, including all vaccine platforms colored in red (Figure 3a–e, right panels). For dogs and minks, all employed vaccines exhibited a survival percentage greater than 80%, and the global survival rates of the whole population were 97.2% and 97.9%, respectively (Figure 3a,c, right panels). Similarly, for the grouped populations of foxes, raccoons, Siberian polecats, and hybrid ferrets WITH Siberian polecats, the survival percentage was greater than 80%, and the global percentage was 100% (Figure 3e, right panel). In contrast, for ferrets, 100% of the animals that received MLVs and chimeric MeV-expressing CDV survived, with a global survival rate of 66.7%, since DNA vaccines were not effective enough (Figure 3b, right panel). Finally, in mice, a peptide-based vaccine resulted in 75% survival; however, the global survival rate was 87.7% (Figure 3d, right panel). Overall, the employed vaccine platforms have demonstrated moderate to high protection against CDV infection, considering that, for all animals, survival in negative vaccination control individuals in populations such as mice and ferrets was 0.0%, and all differences were statistically significant. On the other hand, the MLV CDV vaccine demonstrated approximately 100% protection in all animal populations, indicating the importance of this type of vaccination platform; however, for other alternatives, such as DNA vaccines, their efficacy depends on the animal species (Figure 3a–e, right panels).

## 4. Discussion

Currently, there are different CDV vaccine alternatives for domestic dogs, including MLV vaccines, recombinant viruses, and multivalent vaccines [1,20,79]. This scoping review provides valuable insights into the safety and efficacy of CDV vaccines *in vivo* across domestic and wildlife animal populations, as reported in scientific databases. Through a systematic examination of the literature, this review synthesizes key findings, identifies knowledge gaps, and outlines implications for future research and vaccination strategies, since there are many species that could be affected by CDV, and most of them lack structured research on CDV vaccines.

In total, we included sixty-eight *in vivo* studies on CDV vaccine development (Figure 1) and research, where the United States of America, China, and France had the highest numbers of *in vivo* CDV vaccine trials in domestic and wildlife animals, which coincides with the authors’ affiliations (Figure 2). As reported, collaboration among researchers from different countries seems low in CDV vaccine development, as shown in Figure 2. There is a need for collaborative research in wildlife animal vaccination, new-generation effective and safe vaccines, and population-based immunization strategies to control CDV’s transmission and dissemination. This is essential in advancing scientific knowledge and addressing complex global challenges such as CDV infection for domestic and wildlife animal conservation. By pooling diverse expertise, resources, and perspectives, international collaborations foster innovation and accelerate scientific breakthroughs. Such partnerships enable the sharing of data, methodologies, and best practices, leading to more robust and reproducible findings [93]. Moreover, collaboration across borders enhances the cultural understanding and promotes mutual respect among scientists, laying the foundation for long-term partnerships that transcend geopolitical boundaries [94]. In an increasingly interconnected world, where many scientific questions require multidisciplinary approaches, international collaboration is not only beneficial but also essential in pushing the boundaries of human knowledge and tackling the pressing issues facing human and animal health [95]. Although conducting vaccination studies within wildlife populations is resource-intensive, both financially and logistically, and is influenced by various factors, including the vaccination policy recommendations of the respective countries, there is an urgent need for CDV vaccination in wildlife populations in several biodiverse regions of Asia, South America, and Africa.

Considering that CDV can affect a vast array of domestic and wildlife animals [2,3], there is a small number of species for which there are *in vivo* CDV vaccine studies, which are shown in Table 2, demonstrating the necessity of exploring CDV vaccines for the broad range of wildlife animals affected by CDV. This is crucial as controlling CDV transmission and dissemination in endangered wildlife animal species may contribute to the conservation of these threatened animals [16,96,97]. As expected, most studies were conducted on dogs, ferrets, and mice (Table 2). The presence of only a few studies in other species indicates an essential limitation in CDV vaccine development since there are reports about the transmission of CDV in different species [17]. The circulation of CDV lineages in domestic dogs that have been disseminated only to wild animals has been reported, indicating the importance of controlling CDV infection in domestic and wild animals [15].

There are different commercially available vaccines for CDV infection in dogs, ferrets, and minks based on MLVs and recombinant vaccines [16]. Here, MLV CDV vaccines were studied more frequently than other vaccine alternatives. Notably, recombinant viruses have been widely employed for CDV infection in different animal species (Table 3). Nevertheless, there are some experimental vaccines for CDV based on new alternatives, such as more recombinant viruses, primarily based on the canarypox backbone [26,27,28,29], recombinant mouse adenovirus 1 expressing CDV antigens [35], and formulations based on bacterium-like particles presenting CDV antigens [19]. This review indicates that the development of CDV vaccines has become an important research field since the emergence of geographically distributed CDV lineages has led to new challenges in CDV infection control and dissemination. This is because the current vaccines are based on the Onderstepoort strain, which has not circulated for many years [20] and differs from the current circulating lineages worldwide by more than 10% [14].

On the other hand, it is well known that CDV has a remarkable ability to cross species barriers [5], since the mutations affecting the CDV H protein, which is essential for virus attachment to host cell receptors and the humoral immune response, impair CDV control and facilitate the emergence of novel strains, for which the current vaccines are not completely effective [1]. The presence of antigenic variations among some of the examined CDV wild-type isolates, as well as disparities between these isolates and the vaccine strain Onderstepoort, which is presently employed worldwide, was demonstrated by a cross-neutralization assay, indicating the necessity of developing revised CDV vaccines based on the virus’ genetic and antigenic variations [98].

The safety profile of a vaccine emerges as a focal point when a new vaccine has been developed or tested in other species [99]. While the vaccines reported in this review generally exhibited an acceptable safety profile, varying degrees of adverse reactions, including local injection site reactions, temperature changes, weight loss, behavior changes, and clinical signs, could be reported, as shown in Table 4. The safety and efficacy measures underscore the importance of ongoing vaccine trials, particularly in diverse animal populations, in which the vaccine responses may differ. There are many species for which there are no data about vaccine safety and efficacy, as demonstrated in this review, based on the small number of studies in other species compared to studies in dogs, ferrets, and even minks (Table 2 and Table 4). This is a notable gap identified in this scoping review because of the limited data on CDV vaccine safety and efficacy in wildlife species. Although vaccination efforts have traditionally focused on domestic dogs, wildlife reservoirs play a significant role in CDV’s transmission dynamics [16]. The lack of comprehensive data on vaccine responses in wild animals underscores the urgency of expanding the research efforts in this area. Technical limitations must be overcome, such as the application of vaccination strategies in the field, the use of drones or immunogen bait, vaccine storage and transportation in forest zones, and the monitoring of safety and efficacy, among others, because vaccine evaluation in these animal populations has been possible only in captive animals. Moreover, this scoping review highlights that the humoral immune response has been considered an essential measure of vaccine efficacy, as has been demonstrated for diverse viral infection limitations [100], since most studies have measured nAb (Table 4).

Postimmunization challenge has been employed as an effective measure in vaccine development because it simulates the natural infection protection under controlled conditions with circulating virus strains [19]. As reported in Table 4, approximately half of the included studies investigated CDV challenge in vaccinated animals such as dogs, ferrets, minks, mice, foxes, raccoons, Siberian polecats, and hybrid ferrets x Siberian polecats. Intranasal inoculation, which reproduces the natural CDV infection [1], was the most commonly employed route (Figure 3a–e). Although most employed vaccines have demonstrated moderate to high efficacy, the MLV CDV vaccine is the most effective immunogen in dogs, ferrets, and other evaluated species, as shown in Figure 3, which is consistent with the current literature [20,99]. Moreover, other vaccines, such as DNA plasmids and recombinant viruses, could be employed in animal populations. High survival rates after CDV challenge were demonstrated, in contrast to individuals that were employed as negative vaccination controls, which developed CDD and mostly died (Figure 3a–e, right panels). As with MLV CDV vaccines, recombinant vaccines have been used in different animals, such as dogs, ferrets, minks, and red pandas, to control CDV infection and disease [101,102]. However, commercial recombinant vaccines are based on the Onderstepoort lineage, which reduces the neutralization ability of newly emerged CDV lineages [14]. Although MLVs can induce a strong immune response, attenuated viral strains may not adequately cover the spectrum of CDV variants circulating in diverse geographical regions [16].

One crucial approach to address this evolving scenario is the development of a universal CDV vaccine, as has been proposed for other viruses, such as the *Alphainfluenzavirus influenzae* (Influenza A virus) [103,104], considering the emergence of diverse CDV lineages. The development of a universal vaccine against CDV holds significant importance in mitigating the global impact of this highly contagious and often fatal disease in domestic and wild animals. The current commercially available vaccines offer protection against specific strains of CDV; however, the virus exhibits considerable genetic diversity, leading to vaccine escape and outbreaks among susceptible populations [13,17]. A universal vaccine targeting the conserved regions of the CDV genome could provide broad-spectrum protection against diverse viral strains. Such a vaccine would not only benefit domestic dogs but also contribute to the conservation of endangered wildlife species susceptible to CDV, such as African lions and Amur tigers [18,105].

Through diverse advances in immunology and genomics, researchers are actively exploring novel vaccine candidates capable of eliciting robust and durable immune responses against CDV. The development of a universal vaccine represents a crucial step in safeguarding the health and well-being of canine populations worldwide, as well as protecting vulnerable wildlife species from this devastating viral pathogen [106]. Efficacy assessments have revealed notable variability in vaccine performance across different animal hosts and formulations (Table 4 and Figure 3). Factors such as the vaccine strain, dose, and administration route influence the level of protection conferred against CDV infection [107]. Such variability underscores the complexity of vaccine–host interactions and highlights the need to explore new vaccination approaches. Furthermore, the vaccine efficacy in domestic and wildlife animal species emphasizes the importance of species-specific considerations in vaccine development. Peptide-based vaccines could be a potential alternative [108], because they enable the introduction of multiple immunogenic epitopes. Moreover, their safe profile due to the absence of potential reversion has been considered a notable advantage compared to the inherent risks associated with highly effective live-attenuated vaccine formulations, especially for endangered wildlife animals [109].

This scoping review underscores the imperative need for the design of standardized international trials in prototype species by family or species groups, enabling the validation of the safety and efficacy of the current and prototype vaccine technologies. Emphasis is placed on the necessity of public–private partnerships, as these vaccines have a significantly limited market, rendering them unattractive for investment by commercial enterprises [110]. Additionally, alternative vaccination systems must be developed to reach target species without direct contact with humans, akin to those established for rabies and plague viruses [111,112]; moreover, “transmissible vaccines” have emerged as a novel strategy to augment the reach and protection in wildlife populations [113,114].

Among the limitations of this scoping review were the language restrictions, as the article search was confined to Spanish, English, and Portuguese. Although these languages constitute an essential portion of the scientific production, they do not account for all of it, as studies in Mandarin, German, French, and other languages were excluded. Similarly, the full texts of many articles could not be accessed due to their unavailability. Additionally, the heterogeneity in defining vaccine safety and efficacy and the diversity of CDV vaccine platforms prevented the inclusion of a greater number of articles, since, in some cases, the information could have been confusing. Another important limitation is that the vaccine industry is not required to publish of all its developments and clinical trials in the scientific literature. Therefore, it is plausible that the industrial developmental data of vaccines may not entirely align with the data presented in the current scoping review, despite the existence of animal study guidelines such as GRADE. There are currently no standardized guidelines for the development and evaluation of biological agents, which would allow for the uniformity and systematization of all existing trials across different species. Consequently, significant developments and evaluations in species crucial for conservation efforts could not be included in this scoping review. This limitation arose due to the unavailability of the required information as inclusion criteria in published articles, as exemplified by studies on tigers (*Panthera tigris*) [27], pandas (*Ailuropoda melanoleuca*) [21,115], and red pandas (*Ailurus fulgens*) [101], among others. Moreover, considering that there are no specific repositories in the veterinary sciences for this type of integrative research, this scoping review protocol was not registered. Finally, the survival results combined data from different vaccines, administration routes, and strains used in the immunological challenge. Future studies should conduct meta-analyses of the efficacy considering this variability and heterogeneity.

## 5. Conclusions

In conclusion, this scoping review provides a comprehensive synthesis of the current literature on the safety and efficacy of CDV vaccines *in vivo* in domestic and wildlife animal populations. Our analysis underscores the importance of ongoing vaccine efficacy and safety evaluation, particularly in diverse animal populations affected by CDV, with insufficient vaccine alternatives or studies of commercially available vaccines. While CDV vaccines generally exhibit an acceptable safety profile in dogs, there are significant gaps in vaccine research, particularly in the context of wildlife reservoirs, since little information regarding the current vaccines and new alternatives has been described, although vaccines have shown promise as tools for the control of CDV. Addressing these knowledge gaps through targeted research efforts and collaborative initiatives is imperative for the development of effective vaccination strategies to safeguard animal health and mitigate the impact of CDV on global animal populations, especially for endangered species.

## Figures and Tables

**Table 1:** Studies’ main characteristics.

Year	Country	Species	Individuals	Safety	Efficacy	Challenge	Reference
1958	USA	Dogs	45	T, leukocyte count, and IS daily	nAb	Yes	[37]
1965	USA	Dogs	23	IS daily	nAb	Not	[38]
1965	Germany	Dogs	32	T and CS daily	nAb	Not	[39]
1980	USA	Dogs	8	T, W weekly, and CS daily	nAb	Yes	[40]
1984	USA	Dogs	9	T, W weekly, and CS daily	nAb	Yes	[41]
1986	USA	Dogs	11	T, W biweekly, and CS daily	nAb and sAb *	Yes	[34]
1986	USA	Dogs	8	T and lymphocyte count	nAb and sAb *	Not	[42]
1988	The Netherlands	Dogs	12	T, W, CS, and lymphocyte count	nAb and sAb *	Yes	[43]
1991	USA	Dogs	14	T, W, and CS daily	nAb	Yes	[44]
1992	USA	Dogs	15	T, W, and CS daily	nAb	Yes	[45]
1993	France	Mice	77	Survival	nAb	Yes	[46]
1994	UK	Dogs	36	T, W, and CS daily	nAb	Yes	[47]
1994	USA	Badgers	11	W, and CS daily	nAb	Not	[48]
1995	UK	Mice	48	Survival	sAb *	Yes	[49]
1996	USA	Hyb FxSP	24	T and CS daily	nAb	Yes	[50]
1997	USA	Ferrets	26	T, W, CS, and survival	nAb	Yes	[23]
1997	USA	Dogs	33	Inoculation site and CS daily	nAb	Yes	[51]
1998	France	Mice	58	CS and survival	nAb and sAb *	Yes	[52]
1999	Canada	Raccoons	47	CS local or systemic	nAb	Yes	[53]
1999	USA	Ferrets	37	T, W, CS, and leukocyte count	nAb	Yes	[54]
2000	Switzerland	Dogs	7	T, CS, and leukocyte and lymphocyte count	nAb and N-sAb	Yes	[55]
2000	USA	Dogs	60	CS and survival	nAb	Not	[56]
2000	USA	Ferrets	84	T, W, CS, CDV infection daily, and survival	nAb	Yes	[57]
2001	USA	Ferrets	16	W, CS of CDV infection	nAb	Yes	[58]
2001	Brazil	Dogs	11	CS	nAb	Not	[59]
2001	Finland	Minks, raccoon dogs, silver foxes, and blue foxes	Mink: 40 Raccoon dog: 39 Silver fox: 40 Blue fox: 42	CS	nAb	Not	[60]
2001	Switzerland	Dogs	45	CS	sAb *	Not	[61]
2002	South Africa	Wild dogs	10	CS daily	nAb	Not	[62]
2002	India	Dogs	12	CS and survival	nAb	Not	[63]
2002	USA	Dogs	25	CS and survival	nAb	Yes	[64]
2003	USA	Siberian polecats	29	CS and survival	nAb	Yes	[24]
2003	France	Dogs	9	CS and survival	nAb	Yes	[65]
2004	France	Minks	22	CS and survival	nAb and sAb *	Yes	[66]
2004	Italy	African wild dogs	9	CS	nAb	Not	[67]
2005	USA	Fennec foxes and meerkats	11	CS	nAb	Not	[22]
2007	The Netherlands	Dogs	24	T and CS	nAb	Not	[68]
2007	UK	Ferrets	15	T daily, W weekly, and CS of CDV infection	nAb and sAb *	Yes	[69]
2007	Czech Republic	Ferrets	5	T and CS daily	nAb	Not	[70]
2009	Canada	Ferrets	9	T, W, and CS	nAb and sAb *	Yes	[30]
2009	USA	Southern sea otters	8	CS	nAb and sAb *	Not	[71]
2009	Denmark	Minks	13	CS	nAb	Yes	[72]
2009	Denmark	Minks	10	CS, lymphocyte count, and survival	nAb	Yes	[33]
2012	Denmark	Minks	17	CS	nAb	Yes	[73]
2012	Australia	Dogs	235	CS	nAb	Not	[74]
2012	The Netherlands	Ferrets	24	T, W, CS, and behavior	nAb	Yes	[32]
2013	USA	African wild dogs	21	CS	nAb	Not	[75]
2013	USA	Harbor seals	5	CS local and systemic	nAb	Not	[76]
2014	USA	Wild neartic river otters	22	CS	nAb	Not	[77]
2014	Belgium	Dogs	15	CS	nAb	Yes	[78]
2014	China	Dogs	24	T, W, and CS	nAb	Yes	[79]
2014	Belgium	Dogs	7	T and CS daily	nAb	Yes	[80]
2015	China	Minks	20	T, W, and CS	nAb	Yes	[81]
2016	USA	Cats	20	CS	nAb	Not	[82]
2016	USA	Tigers	102	CS	nAb	Not	[27]
2017	USA	Two-toed sloths	7	CS	nAb	Not	[83]
2017	China	Dogs	15	T, W, CS, and behavior	nAb	Yes	[84]
2019	USA	Dogs	100	W and CS	nAb	Not	[85]
2019	China	Mice	20	W and CS daily	nAb and sAb *	Not	[86]
2019	China	Mice and dogs	Mice: 240 Dogs: 32	T, CS, and survival	nAb and sAb *	Yes	[87]
2019	USA	Red foxes	17	CS	nAb	Not	[88]
2020	China	Mice and foxes	Mice: 96 Foxes: 18	CS and survival	nAb and sAb *	Yes	[26]
2020	USA	Maned wolves	9	CS	nAb	Not	[89]
2020	USA	Tigers and cats	Tigers: 8 Cats: 17	IS daily	nAb	Not	[90]
2021	USA	African wild dogs	10	CS and behavior	nAb	Not	[91]
2022	Belgium	Mice	45	CS	nAb and sAb *	Not	[35]
2022	France	Dogs	25	CS	nAb	Not	[92]
2023	China	Ferrets	20	T, W, CS, and behavior	nAb	Yes	[31]
2023	Canada	Walruses	3	CS	nAb	Not	[28]

* Specific antibodies (sAb) were measured by ELISA; Hyb FxSP: hybrid ferret and Siberian polecat, T: temperature, CS: clinical signs, W: weight, IS: illness signs, nAb: neutralizing antibodies, N-sAb: N-protein-specific antibodies.

**Table 2:** Domestic and wildlife species included in CDV vaccine trials.

Species	*n* ^a^	Percentage (%)	CI 95%	Individuals
Dogs	28	37.3	(26.4–49.3)	892
Ferrets	10	13.3	(6.6–23.2)	236
Mice	7	9.3	(3.8–18.3)	584
Minks	6	8.0	(3.0–16.6)	122
African wild dogs	3	4.0	(0.8–11.3)	40
Tigers	2	2.7	(0.3–9.3)	110
Cats	2	2.7	(0.3–9.3)	37
Others *	17	22.7	(13.8–33.8)	342
Total	75	100		2363

^a^ Number of animal populations evaluated in included studies in the *in vivo* CDV vaccine trials (Table 1). * The other species include badgers, fennec foxes, meerkats, hybrid ferret WITH Siberian polecats, harbor seals, maned wolves, raccoon dogs, silver foxes, blue foxes, raccoons, red foxes, Siberian polecats, southern sea otters, two-toed sloths, walruses, wild dogs, and wild Nearctic river otters, with just one study that employed each of these animal species.

**Table 3:** Characteristics of vaccines used in CDV studies.

		*n* ^a^	Percentage (%)	CI 95%
Vaccine type	MLV CDV	25	32.9	(22.5–44.6)
	Recombinant virus	25	32.9	(22.5–44.6)
	DNA plasmids	13	17.1	(9.4–27.5)
	Inactivated CDV	6	7.9	(3.0–16.4)
	MLV MeV	3	3.9	(0.8–11.1)
	Others *	4	5.3	(1.5–12.9)
	Total	76	100	
Scheme (doses)	One	25	32.5	(22.3–44.1)
	Two	31	40.3	(29.2–52.1)
	Three	16	20.8	(12.4–31.5)
	Four	5	6.5	(2.1–14.5)
	Total	77	100	
Inoculation route	SC	36	39.6	(29.5–50.4)
	IM	30	33.0	(23.5–43.6)
	IN	8	8.8	(3.9–16.5)
	Oral	6	6.6	(2.5–13.8)
	Others **	11	12.1	(6.2–20.6)
	Total	91	100	

^a^ Number of studies that included the vaccine type, scheme, and inoculation route in the *in vivo* CDV trial. Nine studies included more than one vaccine type, eight studies included more than one vaccination scheme, and thirteen studies included two or more inoculation route trials (Table 1). * Chimeric MeV-expressing CDV proteins, ISCOMs, peptide-based vaccines, and purified H and F CDV proteins from only one study. ** Intracranial, intraperitoneal, intradermal, and intravenous. MeV: measles virus, SC: subcutaneous, IM: intramuscular, IN: intranasal.

**Table 4:** Measures of safety, immunogenicity, and viral challenge with route in CDV vaccine studies.

		*n* ^a^	Percentage (%)	CI 95%
Safety	Clinical signs *	65	47.4	(38.9–56.1)
	Temperature	26	19.0	(12.8–26.6)
	Weight loss	21	15.3	(9.7–22.5)
	Survival	14	10.2	(5.7–16.6)
	Behaviors	6	4.4	(1.6–9.3)
	Leukocyte count	5	3.6	(1.2–8.3)
	Total	137	100	
Immunogenicity	nAb	66	81.5	(71.3–89.2)
	Specific Ab ELISA	15	18.5	(10.8–28.7)
	Total	81	100	
Challenge	Yes	37	54.4	(41.9–66.6)
	No	31	45.6	(33.5–58.1)
	Total	68	100	
Challenge Route	IN	22	53.7	(37.4–69.3)
	IV	2	4.9	(0.6–16.5)
	Others **	17	41.5	(26.3–57.9)
	Total	41	100	

^a^ Number of studies that included different strategies for safety and efficacy in the *in vivo* CDV trial. Most studies included more than one safety measure, thirteen studies included both nAb and specific Ab with ELISA, and only two studies included only specific Ab with ELISA. * Clinical signs include symptoms such as dysphagia, rash, apnea periods, hair and skin changes, respiration alterations, and pale conjunctivae, among others, which may be detected during a physical examination. ** IP: intraperitoneal, IM: intramuscular, oculonasal, and oral. IV: intravenous, nAb: neutralizing antibodies for CDV, Ab: antibodies.

## Data Availability

All data are presented in the paper and in the Supplementary Materials. The scooping review was registered at https://osf.io/n9sed (accessed on 22 May 2024) under the https://doi.org/10.17605/OSF.IO/N9SED.

## Supplementary Materials

The following supporting information can be downloaded at: https://www.mdpi.com/article/10.3390/v16071078/s1; Table S1: PRISMA-ScR checklist.
